# What Motivates Young Adults to Talk About Physical Activity on Social Network Sites?

**DOI:** 10.2196/jmir.7017

**Published:** 2017-06-22

**Authors:** Ni Zhang, Shelly Campo, Jingzhen Yang, Petya Eckler, Linda Snetselaar, Kathleen Janz, Emily Leary

**Affiliations:** ^1^ San Jose State University San Jose, CA United States; ^2^ The University of Iowa Iowa City, IA United States; ^3^ Ohio State University Columbus, OH United States; ^4^ University of Strathclyde Glasgow United Kingdom; ^5^ University of Missouri Columbia, MO United States

**Keywords:** physical activity, social marketing, social media

## Abstract

**Background:**

Electronic word-of-mouth on social network sites has been used successfully in marketing. In social marketing, electronic word-of-mouth about products as health behaviors has the potential to be more effective and reach more young adults than health education through traditional mass media. However, little is known about what motivates people to actively initiate electronic word-of-mouth about health behaviors on their personal pages or profiles on social network sites, thus potentially reaching all their contacts on those sites.

**Objective:**

This study filled the gap by applying a marketing theoretical model to explore the factors associated with electronic word-of-mouth on social network sites about leisure-time physical activity.

**Methods:**

A Web survey link was sent to undergraduate students at one of the Midwestern universities and 439 of them completed the survey.

**Results:**

The average age of the 439 participants was 19 years (SD=1 year, range: 18-24). Results suggested that emotional engagement with leisure-time physical activity (ie, affective involvement in leisure-time physical activity) predicted providing relevant opinions or information on social network sites. Social network site users who perceived stronger ties with all their contacts were more likely to provide and seek leisure-time physical activity opinions and information. People who provided leisure-time physical activity opinions and information were more likely to seek opinions and information, and people who forwarded information about leisure-time physical activity were more likely to chat about it.

**Conclusions:**

This study shed light on the application of the electronic word-of-mouth theoretical framework in promoting health behaviors. The findings can also guide the development of future social marketing interventions using social network sites to promote leisure-time physical activity.

## Introduction

### Background

As of 2015, 90% of Internet users in the United States between the ages of 18 and 29 years use social network sites (SNSs) [1]. SNSs allow individuals to share their social connections and provide a platform for peer communication in their daily lives [2], offering great potential to influence a large number of young adults’ health choices. For example, young adults liked receiving personalized health information about the HPV vaccine from peers on SNSs [3]. Further, social support from peers on SNSs can indirectly increase college students’ intentions to participate in leisure-time physical activity (LTPA) [4]. Here, LTPA refers to a specific domain of physical activity in which people engage voluntarily during their free time because of physical activity has the potential to provide feelings of enjoyment, control, or mastery [5,6].

Engaging users to generate and share content is crucial to the outreach and success of health promotion activities that use SNSs [7]. Peer communication on SNSs can increase the reach of health information exponentially if forwarded consecutively by many people [8]. However, little is known about what motivates people to actively initiate peer communication about health topics on their own pages/profiles, where health information can be seen by all their contacts, rather than only by members of certain SNS health groups. This exploratory study applies a marketing theoretical model to fill this gap in understanding.

In marketing, online peer communication with “positive or negative statements made by potential, actual, or former customers about a product or company, which is made available to a multitude of people and institutions via the Internet” is referred to as electronic word-of-mouth (eWOM) [9]. eWOM on SNSs has been perceived by customers as a more reliable source of product information than the corporate-sponsored messages in traditional marketing, such as advertising, sales promotions, and public relations [10,11]. There are two components of eWOM: opinion providing and opinion seeking [12]. *Opinion providing* is the *process* by which some individuals influence the attitudes or behaviors of others [12]. Similarly, *opinion seeking* is the *process* by which people seek advice about a product [12,13]. In social marketing, the product can be the health behavior of the individual [14].

**Figure 1:**
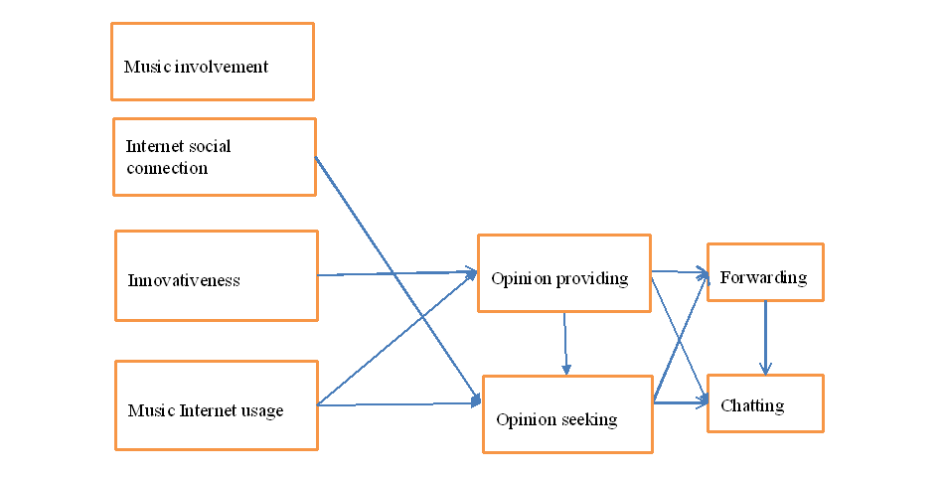
EWOM (electronic word-of-mouth) path model.

In this study, we focus on one specific outcome or product of a health behavior: LTPA. To systematically study the factors that affect the different components and formats of eWOM about LTPA, we applied a marketing theoretical framework, *the Path Model of the Antecedents and Consequences of Online Word-of-Mouth (hereafter the eWOM Path Model)* [12]. The eWOM Path Model is demonstrated in Figure 1 [12].

### Antecedents of eWOM

Based upon the eWOM Path Model [12] and previous marketing literature [15], the first predictor of eWOM we investigated is involvement, which refers to a person’s perception of a product’s relevance, here LTPA, based on inherent needs, values, and interests. Product involvement, or personal interest in the product, or the excitement resulting from product ownership and use, motivates people to initiate traditional word-of-mouth (WOM) about commercial products [16]. Previous research suggests that involvement is associated with traditional WOM and eWOM [12,17]. Here, the role of LTPA involvement in eWOM, specifically on SNSs, is investigated.

Furthermore, this study distinguishes between two kinds of involvement: affective and cognitive. In marketing research, affect refers to “a class of mental phenomena characterized by a consciously experienced, subjective feeling state, commonly accompanying emotions and moods” [18]. Aligned with this description, this study defines affective involvement in LTPA as relevance to LTPA resulting from a consciously experienced, subjective feeling state, commonly accompanying emotions and moods. Correspondingly, cognitive involvement in LTPA can be defined as relevance to LTPA with respect to the cognitive attribution of LTPA. This research investigates if affective involvement, cognitive involvement, or both predict eWOM about LTPA.

Applying the eWOM Path Model [12] in the context of SNSs, general Internet social connection is replaced with two indicators of social connections on SNSs. The first indicator is perceived strength of ties to all contacts. With eWOM, participants engage in public communication with a network of people [19] and this makes one-to-one social ties less relevant in eWOM than in traditional WOM [20]. Thus, we regard all contacts on SNSs as a holistic network and investigate if perceived strength of ties to all contacts on SNSs is a predictor of eWOM about LTPA on SNSs.

To further scrutinize the network, this study examines another indicator of social connections, which is the ratio of strong ties on SNSs. For traditional WOM, strong ties often refer to close contacts like family and close friends, whereas weak ties include relationships with acquaintances [21,22]. Similarly, for eWOM, strong ties often refer to family members, relatives, and close friends, whereas weak ties include acquaintances, classmates, and neighbors [23]. For traditional WOM, referrals are more likely to happen between strong ties, which are also considered as more influential for the product [24]. However, for eWOM on SNSs, information can reach audiences with both strong and weak ties simultaneously. Thus, we hypothesize that people with larger numbers of strong ties are more likely to talk about LTPA on SNSs.

Applying the eWOM Path Model to study eWOM about online music, Sun and colleagues [12] found that people who actively use the Internet for music consumption were more likely to provide and seek opinions about online music. In the context of LTPA, the consumption of the product is in the form of participating in LTPA, which cannot be done online. Moreover, according to the literature [15], self-enhancement, which refers to the tendency to seek experiences that improve or bolster the self-concept by drawing attention to one’s skills and talents [25], was one main antecedent of eWOM about a commercial product. Thus, we will investigate this relationship in the context of LTPA and hypothesize that people who participate in more LTPA are more likely to talk about it on SNSs as a means of self-enhancement.

### Opinion Providing and Opinion Seeking

These are two distinguished components of eWOM: opinion providing and opinion seeking [12]. Adapted from a marketing context, here *opinion providing* is the *process* by which some individuals influence others’ LTPA participation. Similarly, *opinion seeking* is the *process* by which people seek advice when participating in LTPA [12,13]. This study explores various antecedents for these two components of eWOM respectively, in order to guide future interventions that encourage young adults to provide or seek opinions of LTPA.

### Formats of eWOM

There are two formats in eWOM: chatting and forwarding [12]. Chatting is referred to as creating posts using one’s own words [3,12]. eWOM on SNSs can be forwarded directly by reposting exact quotes from other contacts and posting existing URLs. In the eWOM Path Model [12], forwarding was the predictor of chatting.

Sun and colleagues [12] found that both opinion providing and seeking were predictors of chatting and forwarding about online music respectively. In a qualitative study on eWOM about HPV vaccine on Facebook, female college students revealed their preference for forwarding rather than chatting in general, because of their lack of HPV vaccine knowledge [3]. Applying the eWOM Path Model in the context of eWOM on SNSs about LTPA, this study investigates whether people who provide opinions are more likely to chat or forward information about LTPA on SNSs and whether people who seek opinions about LTPA on SNSs are more likely to chat or forward information about LTPA on SNSs.

Furthermore, Sun and colleagues [12] found that providing online opinions predicted online opinion-seeking. They speculated that people who are more likely to provide opinions are more likely to seek opinions in order to update their information and knowledge. In the context of eWOM on SNSs, one study found that providing opinions about dining is a predictor of seeking dining-related information on SNSs [26]. This study investigates if the same association exists in the context of LTPA.

**Figure 2:**
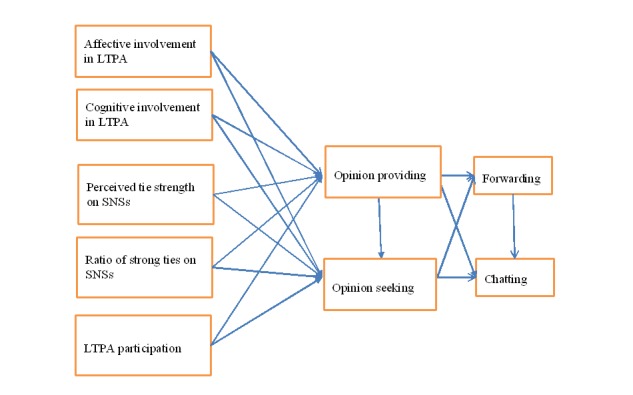
Conceptual framework.

In summary, this study applies the eWOM Path Model to LTPA to examine the factors associated with two components of eWOM, opinion providing and opinion seeking, by using two formats of eWOM: forwarding and chatting. The conceptual framework for this study is demonstrated in Figure 2.

## Methods

### Data Collection

A mass recruitment email was sent to all undergraduate students enrolled at one of the Midwestern universities in the fall of 2011. In order to prevent self-selection due to participation in LTPA, the topic of LTPA was not mentioned in the recruitment email. A link to the Web survey was provided in the email. Participants were compensated with entry into a lottery in which US $10 gift cards were given to 50 participants. Participation was anonymous and this study was approved by the university’s Institutional Review Board. In total, 439 participants out of all the undergraduate students between the age of 18 and 24 completed the survey within one month.

### Measurements

#### Antecedents of eWOM

##### Affective Involvement

Participants were asked to rate four items adapted from Zaichkowsy’s [27] Personal Involvement Inventory: “To me, participating in leisure-time physical activities is (1) ‘boring-interesting,’ (2) ‘exciting-unexciting,’ (3) ‘appealing-unappealing,’ or (4) ‘fascinating-mundane’” on a 5-point (from 1 to 5) semantic differential scale. Items (2), (3), and (4) were reversely coded before composite scores were summed, where higher scores indicated higher affective involvement. The Cronbach alpha value was .90.

##### Cognitive Involvement

Participants were asked to rate six items adapted from Zaichkowsy’s [27] Personal Involvement Inventory “To me, participating in leisure-time physical activities is (1) ‘unimportant-important,’ (2) ‘irrelevant-relevant,’ (3) ‘means nothing-means a lot to me,’ (4) ‘worthless-valuable,’ (5) ‘involving-uninvolving,’ or (6) ‘not needed-needed’” on a 5-point (from 1 to 5) semantic differential scale. Item (5) was reversely coded before composite scores were aggregated, where higher scores indicated higher cognitive involvement. The Cronbach alpha value was .88.

##### Perceived Strength of Ties

Perceived strength of ties was assessed using three questions [28]. Participants were asked to rate how frequently they individually communicated with all their contacts on their most-used SNS on a scale of 1 to 7 (never-very frequently). Participants were then asked to rate the importance of all of their contacts on this SNS, ranging from 1 to 7 (not at all important-very important). Afterwards, participants rated how close they individually felt to all their contacts on this SNS, ranging from 1 to 7 (not at all close-very close). The Cronbach alpha value for perceived strength of ties was .70; composite scores were summed and higher scores indicated higher perceived strength of ties.

##### Perceived Ratio of Strong Ties

Perceived ratio of strong ties was assessed using two questions [23]. The first question asked “Approximately how many total contacts do you have on this SNS?” For Facebook/MySpace and other SNSs besides Twitter, “contacts” refers to “friends.” For Twitter, “contacts” refers to “followers.” A follow-up question asked, “How many contacts in each category (parents/guardians, siblings, other family members, and close friends) do you have on this SNS?” The ratio of strong ties was calculated by dividing the sum of parents/guardians, siblings, other family members, and close friends by the number of all contacts on this SNS. Higher scores indicated a higher perceived ratio of strong ties.

##### LTPA Participation

A physical activity checklist consisting of 27 types of LTPA was adapted from the Physical Activity Questionnaire for Adults to measure LTPA participation in the past 7 days [29]. For each type of LTPA, participants were asked to indicate (1) whether they participated in it and (2) how many times they had participated in it over the previous seven days. Participation counts for each type of LTPA over the previous seven days were categorized into five groups: (1) 0=none; (2) 1=1-2 times; (3) 2=3-4 times; (4) 3=5-6 times; (5) 4=7 times or more. LTPA participation was measured using the mean score calculated by dividing total participation counts for all types of LTPA by the number of types of LTPA. Higher LTPA participation scores indicated increased participation.

#### Components of eWOM

##### Opinion Providing About LTPA

The 5-point Likert scale ranging from “strongly disagree” to “strongly agree” used by Flynn et al [13] for opinion leaders was adapted in this study. The following is an example of one of the three items: “My contacts on this SNS pick a leisure-time physical activity based on what I have told them on the SNS.” The Cronbach alpha value was .89. The composite scores were summed.

##### Opinion Seeking About LTPA

The 5-point Likert scale ranging from “strongly disagree” to “strongly agree” for opinion seeking developed by Flynn et al [13] was adapted in this study. A representative example, one of the three items, is “When considering participating in a leisure-time physical activity, I ask my contacts for advice on this SNS.” Composite scores were summed. The Cronbach alpha value was .84.

#### Formats of eWOM

##### Forwarding About LTPA on This SNS

The measurement contained six items with a 5-point Likert scale, ranging from “strongly disagree” to “strongly agree” [12]. One item was, “I tend to use the ‘Share this site’ function to share websites on this SNS when I find information about leisure-time physical activity.” Composite scores were summed for each item. Higher scores indicated that participants forwarded more information about LTPA on their preferred SNS. The Cronbach alpha value for forwarding was .92.

##### Chatting About LTPA on This SNS

The chatting measure contained five items with a 5-point Likert scale, ranging from “strongly disagree” to “strongly agree” [12]. One item was, “On this social network site, I like to share information with my contacts about my favorite leisure-time physical activity.” Composite scores were summed. Higher scores indicated that participants shared more about LTPA with their SNS contacts. The Cronbach alpha value was .91.

#### Covariates

##### Demographics

Participants provided their age (between 18 and 24), gender (male or female), and race/ethnicity (non-Hispanic white or other).

#### Data Analysis

Descriptive analyses were conducted using SPSS version 22 (IBM Corp). Spearman rank correlations were tested among the antecedents, components, and consequences of eWOM. Path models with 439 cases were conducted using AMOS version 22 (IBM Corp), a structural equation modeling program. AMOS has been widely used in other health communication studies [30]. Diagnostic analyses were performed to test the assumptions of the path model analysis. Outliers (n=22) were deleted and the square root transformation was used for the ratio of strong ties.

Comparative fit index (CFI) and the root mean square error of approximation (RMSEA) were used to estimate the model fit. According to Kline [31], a CFI value over .90 and RMSEA value between .05 and .08 indicate an acceptable model fit [31]. Multi-group analyses were conducted to test the difference between groups based on sex and ethnicity, respectively. No significant differences were observed. Age was not significantly associated with any of the eWOM components and consequences in the path model. Thus, the final path model did not include these covariates. The path coefficients were standardized regression coefficients with *P*<.05, indicating significant associations.

## Results

### Demographics

The average age of the 439 participants was 19 years (SD=1 year, range: 18-24). Just over 79.0% (343/434) of the participants were female and the majority of the participants were non-Hispanic whites (81.0%%, 354/437). More than 77.9% (341/438) of the participants indicated that they visited their SNS several times a day.

### Bivariate Analysis Results

Spearman correlation matrix is shown in Table 1 and includes only 394 cases as 45 cases, with missing values for at least one of these variables, were deleted list-wise. These correlations provide pair-wise associations between the variables and give an indication of potential relationships in the path model.

**Table 1:** Spearman correlation, mean, and standard deviation for variables of interest.

N=394	1	2	3	4	5	6	7	8	9	Mean	SD
1. Affective involvement	1.00									15.26	3.89
2. Cognitive involvement	.73									25.31	4.41
3. Perceived strength of ties	.06	−.02								11.73	3.54
4. Ratio of strong ties	.06	.05	.18^a^							18.02	25.46
5. LTPA participation	.31^a^	.32^a^	−.06	.02						1.86	1.44
6. Opinion providing	.23^a^	.18^a^	.27^a^	−.00	.09					6.56	3.26
7. Opinion seeking	.12*	.04	.29^a^	−.00	−.01	.53^a^				5.78	3.01
8. Forwarding	.13^a^	.08	.27^a^	−.01	.09	.54^a^	.62^a^			10.54	5.45
9. Chatting	.25^a^	.19^a^	.34^a^	.06	.10^b^	.68^a^	.71^a^	.70^a^	1.00	10.69	5.15

^a^Correlation is significant at the 0.01 level (2-tailed).

^b^Correlation is significant at the 0.05 level (2-tailed).

### Path Analysis Results

**Figure 3:**
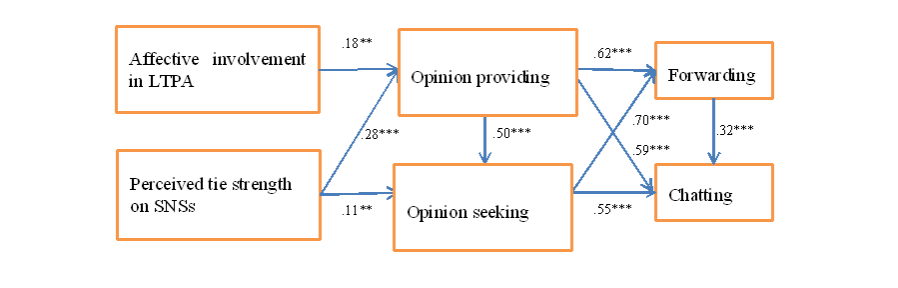
Path model with significant path coefficients only.

Statistically significant relationships were observed between perceived strength of ties and both opinion providing and opinion seeking, whereas cognitive involvement was associated with opinion providing only (see Table 2). Forwarding and chatting behaviors were also associated with opinion providing and seeking behaviors (see Table 2). The path model produced a good fit (RMSEA=0.054; CFI=0.990; χ^2^_10_=22.1, *P*=.01). Path coefficients are displayed in Table 2, while Figure 3 illustrates only significant paths.

**Table 2:** Parameter estimates for causal paths.

Hypotheses	Causal Paths^a^	Path coefficient estimates^b^	Standard error^b^	*P* value^b^
H1a	Affective involvement →Opinion providing	.175	.058	.003
H1b	Affective involvement →Opinion seeking	.058	.049	.24
H2a	Cognitive involvement →Opinion providing	.018	.052	.73
H2b	Cognitive involvement →Opinion seeking	−.080	.044	.07
H3a	Perceived strength of ties →Opinion providing	.281	.043	<.001
H3b	Perceived strength of ties →Opinion seeking	.110	.038	.004
H4a	Ratio of strong ties →Opinion providing	−.054	.068	.43
H4b	Ratio of strong ties →Opinion seeking	−.040	.057	.49
H5a	LTPA participation →Opinion providing	−.051	.106	.63
H5b	LTPA participation →Opinion seeking	−.099	.089	.26
H6a	Opinion providing →Forwarding	.642	.083	<.001
H6b	Opinion providing →Chatting	.591	.059	<.001
H7a	Opinion seeking →Forwarding	.702	.087	<.001
H7b	Opinion seeking →Chatting	.501	.063	<.001
H8	Opinion providing →Opinion seeking	.497	.043	<.001
H9	Forwarding → Chatting	.317	.034	<.001

^a^Goodness-of-fit statistics: CFI=0.990; RMSEA=0.054.

^b^Results controlled for age, gender, race/ethnicity, length of SNS membership, and frequency of using SNSs.

Only affective involvement was significantly associated with online opinion providing (*P*=.003). Perceived tie strength was significantly associated with both online opinion providing (*P*<.001) and opinion seeking (*P*=.004), as expected. As outlined in the path model, both online opinion providing and online opinion seeking were significantly associated with forwarding (*P*<.001 for both) and chatting (*P*<.001 for both). Online opinion providing is significantly associated with online opinion seeking (*P*<.001). Additionally, forwarding was significantly associated with chatting (*P*<.001).

## Discussion

### Principal Findings

This study bridges a gap in the literature by exploring the antecedents and consequences of eWOM about LTPA on SNSs among traditionally-aged (18-24 years) college students. The study observed that students with high affective involvement and stronger perceived strength of ties on SNSs were more likely to provide information and/or opinions about LTPA. However, cognitive involvement and the ratio of strong ties were not significantly related to higher opinion providing. The study supported the ideas that forwarding and chatting were two consequences for both opinion providing and opinion seeking.

### Antecedents of eWOM

This was one of the first studies that differentiated between affective and cognitive involvement in the role of predicting opinion providing and opinion seeking. In studies on traditional WOM, different kinds of involvements, such as product-involvement, self-involvement, other-involvement (eg, to “share” the pleasure), and message-involvement have been documented [32]. Previous research on eWOM has, so far, not distinguished between the various kinds of involvement [12].

This study revealed the roles that these two types of involvement play in predicting eWOM. Only affective involvement, and not cognitive involvement, in LTPA was a predictor of opinion providing on SNSs. This suggests that, if students found a certain LTPA interesting, exciting, appealing, and/or fascinating, they were more likely to share the experience on SNSs. The importance of affective involvement is supported by one study, which found that students’ top motives for sending pass-along emails were “because it’s fun, and because they enjoyed it, because it’s entertaining” [33]. Similarly, in e-commerce, the effect of affective involvement on purchase intention was nearly double that of cognitive involvement [34]. It is worth noting that the described affect is a positive one here, in that participants described positive emotions that arose from engaging in LTPA, which in turn led to providing their opinion. This is a well-known path to generating eWOM, as past research shows that the main motivation to share information online is because people are positively involved with a brand [35].

Neither affective nor cognitive involvement predicted opinion seeking. This could be explained by the difference between LTPA and other traditional commercial products. As is not the case with these products, individual LTPA participation could be considered more personal, fueling needs for specific opinions and information, which may be communicated offline or through private online messages. Alternatively, due to its experiential nature, LTPA could lead to different experiences for different people because of the various physical capabilities, accessibility to the LTPA, and so on, whereas purchasing a product generates a more uniform response. Past research has shown that experiential eWOM comments are seen as less helpful than utilitarian ones, thus possibly driving online users not to seek them in the first place [36].

Adapting to the SNSs context, this study investigated two indicators of strength of ties, perceived strength of ties, and ratio of strong ties and found different effects for each on eWOM. SNS users who perceived strong ties with all their contacts on this SNS were more likely to provide or seek opinions about LTPA. In a previous study, only opinion seeking was influenced by strong ties [12]. Context may play a role in these superficially different results as physical activity could be considered a more personal experience than music. Thus, users might feel more comfortable providing opinions about LTPA when they feel closer to all their contacts on SNSs because of the personal nature of LTPA experiences.

Consistent with previous work [37], perceived ratio of strong ties in users’ social networks on the SNSs was not a predictor for any of the eWOM components in this study. This could be explained by the idea that users use more privacy-related functions (eg, sharing photos) to communicate with close friends on Facebook [38]. Our findings imply that users talk about LTPA on SNSs regardless of the number of close friends.

Surprisingly, LTPA participation was not related to either component of eWOM—opinion providing or opinion seeking. These results are inconsistent with a major motivation to use SNSs: self-presentation [39]. SNSs allow users to construct their identities through pictures, profile information, and wall content [40]. When examining eWOM about physical activity on Twitter, one study found that 38% of the tweets about LTPA were about users’ previous and current LTPA participation [41]. One explanation could be that there are different channels for opinion providing and opinion seeking for someone who participates in LTPA. The Google/Keller Fay group [42] found that the predominant method of word-of-mouth is still offline. Therefore, it is possible that those who participate in LTPA would communicate with their friends through other channels such as face-to-face, phone calls, or text messaging, rather than through eWOM on SNSs. Further, this result shows that positive emotional involvement with LTPA is more important for sharing opinions than actual participation in LTPA.

### Consequences of eWOM

This study found that both chatting and forwarding were consequences of increased opinion providing and opinion seeking about LTPA on SNSs. SNS users who were more likely to provide opinions about LTPA on SNSs were also more likely to seek opinions and information. Additionally, users who were more likely to forward information about LTPA were more likely to chat about it too. Students with either increased opinion providing or opinion seeking were more likely to either forward information about LTPA or chat about it. . These results are consistent with the findings of Sun and colleagues on music-related eWOM [12].

This study provides the first empirical evidence for adapting the eWOM Path Model [12] to a heath context. These findings can guide future interventions using eWOM on SNSs to promote physical activity and other health behaviors. SNS users who have positive emotional connections to LTPA and who feel closely connected to all their contacts can be recruited to be peer leaders to disseminate information about LTPA. Further, public health practitioners should emphasize the “fun” part of LTPA classes or programs to harness the potential for eWOM on SNS and increase participation. Examples might include, “Even if you have never danced before, you will have fun and feel successful in a Zumba class!” [43].

In terms of future research, this study provides the foundation for examining the antecedents and formats of health-related eWOM, which has the highest potential for being shared or discussed. One of the values of eWOM is its organic spread among networks of users or, in other words, its viral aspect. Certain messages (eg, positive emotional ones) may have a higher viral potential than others and future research can examine this further, which will benefit health marketers by harnessing users’ natural tendencies to share information and enabling them to motivate one another into healthier behaviors.

The study population was limited by self-selection; however, efforts were made to decrease the selection bias through careful wording in the recruitment email. Specifically, in an effort to reach those who do not participate or are not interested in LTPA, the LTPA focus of the study was not mentioned in the recruitment email survey introduction. However, the study sample may not be representative of the population of undergraduate students at one of the midwestern universities. Furthermore, we only included participants at traditional college, aged between 18 and 24. Study respondents tended to be younger, freshmen, living on campus, and female, as compared with the population of Midwestern University students. Thus, caution is required when applying the results to a larger population.

Another limitation of this study is associated with self-reporting. Although the online questionnaires were filled anonymously, the possibility of social desirability to over-report the values of certain variables, such as the LTPA and opinion providing about LTPA on SNSs, cannot be ruled out.

### Conclusions

This study contributes to the existing literature by examining the antecedents and formats of eWOM about LTPA on SNSs, applying a theoretical framework typically used in marketing. Results indicate that young adults who feel that LTPA is interesting and fun and are closer to all of their contacts on SNSs, are more likely to provide opinions and information about LTPA on SNSs. Those who provide opinions and information are also more likely to seek opinions and information from others. Those who forward information about LTPA are more likely to talk about LTPA using their own words. Future interventions using SNSs to spread eWOM about LTPA should increase young adults’ affective involvement in order to encourage their organic comments to other users. Young adults who are more affectively involved in LTPA and feel closer to all their contacts on SNSs could be recruited to be peer leaders to provide opinions and information. Future interventions can also encourage young adults who forward information about LTPA on SNSs to chat about it too, as that is a more engaged behavior, which may motivate them or others into participation in physical activity.

## Abbreviations

eWOM: electronic word-of-mouth
LTPA: leisure-time physical activity
SNS: social network sites
WOM: word-of-mouth
